# Supplementary material for: Misic, a general deep learning-based method for the high-throughput cell segmentation of complex bacterial communities
Source: eLife. 2021 Sep 9;10:e65151. doi: 10.7554/eLife.65151 (PMC8478410; doi:10.7554/eLife.65151)
Supplement: Supplementary file 1. [file elife-65151-supp1.pdf]

Table S1. Bacterial strains used in this study

Strain name	Strain/Genotype	Origin
Myxococcus xanthus		
DZ2		Laboratory collection
DM14	DZ2 *attmx8 ftsZ*-NG	This work
DM31	DZ2 p3068-sfGFP	This work
Escherichia coli		
MG1655		Laboratory collection
EC500	TOP 10 pFPV-mCherry (pGG2-rpsM-mCherry)	Laboratory collection
Pseudomonas aeruginosa	Wild type strain PAK	Laboratory collection
Bacillus subtilis	Prototrophic wild-type strain, 168CA *trpC*+	Laboratory collection
Bacillus subtilis	Wild type, Strain 168	Laboratory collection
Caulobacter crescentus	NA1000-*hfsA*+	Marks M.E et al. J. Bact. 2010[15]
Anabaena nostoc	PCC 7120	Pasteur Institute cyanobacterial collection
Desulfovibrio vulgaris	Wild type strain	Laboratory collection
