# Supplementary material for: Misic, a general deep learning-based method for the high-throughput cell segmentation of complex bacterial communities
Source: eLife. 2021 Sep 9;10:e65151. doi: 10.7554/eLife.65151 (PMC8478410; doi:10.7554/eLife.65151)
Supplement: Supplementary file 2. [file elife-65151-supp2.pdf]

Table S2. Primers

Primer name	Sequence	Use	Construct
oDM1	CTC-TAG-AAC-TAG-TGG-ATC-CGA-ACA-ACC-GCC-GCG-TGG-GG	cloning of *ftsZ*	pDM6
oDM2	GTA-TTT-CAC-ACC-GCA-TAT-GTT-ACG-GCA-GTT-CCG-TCT-GGC	cloning of *ftsZ*	pDM6
oDM16	cct-gca-ggt-cga-ctc-tag-atc-act-tat-aga-gtt-cat-cc	cloning of Neon-Green	pDM6
oDM17	gac-gga-act-gcc-ggg-tac-cgg-tac-cgg-gcc-ccc-cct-c	cloning of Neon-Green	pDM6
oDM53	GTT-CTT-CAC-CTT-TAG-ACA-TTG-ACA-CTC-CTC-AAA-AAT-AAA-TGG-A	cloning of *MXAN_3068* upstream region	pDM14
oDM54	GGG-GAT-CCG-GGC-GAA-CGG-GAA-TTC-TA	cloning of *MXAN_3068* upstream region (p3038)	pDM14
oDM61	CCG-CAT-ATG-TTA-TTT-GTA-GAG-CTC-ATC-C	cloning of sfGFP downstream of p3068	pDM14
oDM62	TTT-TGA-GGA-GTG-TCA-ATG-TCT-AAA-GGT-GAA-GAA-C	cloning of sfGFP downstream of p3068	pDM14
