# Supplementary material for: Misic, a general deep learning-based method for the high-throughput cell segmentation of complex bacterial communities
Source: eLife. 2021 Sep 9;10:e65151. doi: 10.7554/eLife.65151 (PMC8478410; doi:10.7554/eLife.65151)
Supplement: Supplementary file 3. [file elife-65151-supp3.pdf]

**Table S3: Plasmids**

| Plasmid name | Backbone | Genotype |
|---|---|---|
| pDM6 | pKA32 (Treuner-Lange, Mol. Micro 2013) | Pnat-*ftsZ*-linkerNeonGreen |
| pDM14 | pSWU19 | pSWU19_*p3068*-sfGFP |
